# PREFRONTAL CORTICAL ACTIVITY DIFFERENCES WHILE DUAL-TASK WALKING IN OLDER ADULTS WITH IMPAIRED MOBILITY

**DOI:** 10.1093/geroni/igz038.2922

**Published:** 2019-11-08

**Authors:** Manuel E Hernandez

**Affiliations:** University of Illinois at Urbana-Champaign, Urbana, Illinois, United States

## Abstract

Mobility impairments are prevalent in older adults. Whereas walking had traditionally been viewed as an autonomous process, evidence over the last decade has shown that cognitive processes such as attention and executive function have a significant impact on gait function in older adults. However, the exact neural mechanisms underlying difficulties in the control of mobility in older adults remains an open question. We examine the changes in the executive control of mobility in older adults with mobility impairments using functional near-infrared spectroscopy, as operationalized by performance in the community balance and mobility scale (CB&M). We hypothesized that prefrontal cortical (PFC) activity increases would be higher in older adults with mobility impairments, compared with older adults without mobility impairment, as dual-task walking difficulty increased. Older adults with (n=10, mean±SD age: 77±8 years, 8 females, CB&M= 58±12) and without mobility impairment (n=14, mean±SD age: 63±9 years, 11 females, CB&M= 87±6) were recruited from the local community. Dual-task walking was performed at a comfortable pace, while the difficulty of the concurrent cognitive task was increased using the modified Stroop test. PFC activity was measured using measures of oxygenated hemoglobin across the PFC. Older adults with mobility impairments demonstrated disproportionate increases in PFC activity, in comparison to those without mobility impairments, as the difficulty of the concurrent cognitive task increased (P<.001), even after controlling for age. In conclusion, these data suggest that older adults with mobility impairments may require greater attentional resources than those without mobility impairments when concurrently performing thinking and walking tasks.

